# Validation of an adapted Italian-language version of the Sociocultural Attitudes Toward Appearance Questionnaire-3 (SATAQ-3), within a female population: the Sociocultural Attitudes Toward Appearance Questionnaire - Social Media (SATAQ-SM)

**DOI:** 10.3389/fpsyg.2023.1193062

**Published:** 2024-04-16

**Authors:** Anna Maria Riccardo, Giulia Ferrazzi, Sara Catellani, Anna Maria Gibin, Anna Maria Nasi, Mattia Marchi, Gian Maria Galeazzi, J. Kevin Thompson, Luca Pingani

**Affiliations:** ^1^Department of Biomedical, Metabolic and Neural Sciences, Università degli Studi di Modena e Reggio Emilia, Modena, Italy; ^2^Direzione delle Professioni Sanitarie, Azienda USL-IRCCS di Reggio Emilia, Reggio Emilia, Italy; ^3^Dipartimento ad Attività Integrata Salute Mentale e Dipendenze Patologiche, Azienda USL-IRCCS di Reggio Emilia, Reggio Emilia, Italy; ^4^Department of Psychology, University of South Florida, Tampa, FL, United States

**Keywords:** social media, sociocultural influence, body image, SATAQ-3, SATAQ-SM

## Abstract

Sociocultural Attitudes Towards Appearance Questionnaire-Social Media (SATAQ-SM) is a self-administered questionnaire for the evaluation of social media pressure and internalization of beauty standards. This study aims to validate the SATAQ-SM an adapted Italian version of the Sociocultural Attitudes Towards Appearance Questionnaire third version (SATAQ-3). Confirmatory factor analysis was used to investigate whether the empirical data fitted the four-factor structure of SATAQ-3. Assessment of goodness-of-fit was based on standard model fit criteria: relative χ^2^ value (χ^2^/df), Root Mean-Squared Error of Approximation (RMSEA), Comparative Fit Index (CFI) and Tucker–Lewis Index (TLI). Internal consistency was assessed using McDonald’s omega. Criterion validity was calculated by correlating the SATAQ-SM factors scores with the total score of the Rosenberg self-esteem scale (RSES) and Eating Attitudes Test (EAT-26). Four-hundred and eighty-five females agreed to participate in the study. The four-factor model appears to be confirmed by the fit indices: χ^2^/df = 3.73, RMSEA = 0.07, CFI = 0.99 and TLI = 0.99. All the items defining the four factors had a factor loading of ≥0.40. McDonald’s omega of the entire questionnaire was equal to 0.95 and for the four subscales it did not assume values lower than 0.81. The correlations between the factor score of SATAQ-SM and the RSES were all negative and statistically relevant (*p* < 0.001); the correlations between the scores of the SATAQ-SM subscales and the total score of the EAT-26 are all positive and statistically significant. SATAQ-SM demonstrated good psychometric properties to assess the influence of social media on body image perception related to social media.

## Introduction

Numerous definitions have been stated for “body image”: Paul Schidler defined it as “the image and appearance of the human body that we form in the mind, that is, the way our body appears to us” (Schilder, 1935; Posavac and Posavac, 2002). Instead, according to Peter Slade, “the subjective image we have in our mind of the shape, size and feelings” is the real representation that each person has of his own body (Slade, 1988). The National Eating Disorders Collaboration says that body image is a combination of the thoughts and feelings that you have about your body (National Eating Disorders Collaboration, 2022).

Body image concept is tightly linked to body dissatisfaction, which, in turn, is closely related to eating behavior disorders (Aparicio-Martinez et al., 2019; Andersen and Swami, 2021; Jiménez-Limas et al., 2022). Moreover, body dissatisfaction can have implications on a person’s health as it is associated with depression, low self-esteem, anxiety, tobacco use, self-harm, alcohol and other substance abuse (Kostanski and Gullone, 1998; Baker et al., 2018; Bornioli et al., 2019).

The development of problems related to body image and eating disorders can be explained by the tripartite model of influence (Thompson et al., 1999; Keery et al., 2004) which identifies peers, parents, and mass media as primary sources of influence. According to the literature, these three sources of influence reinforced the “ideal of beauty,” emphasizing thinness for women and muscles for men (Griffiths et al., 2013). Sociocultural theory posits that influence is mediated by two factors: the internalization of beauty ideals and physical appearance comparisons (Thompson et al., 1999). Internalization occurs when an individual accepts society’s beauty ideals and adopts behaviors to attain that ideal (Thompson et al., 1999). This theory further suggests that the internalization of unattainable ideals, combined with ongoing physical appearance comparisons, can negatively impact body satisfaction (Thompson et al., 1999).

It has been shown that exposure to traditional media (television, cinema, and magazines) is strongly linked to body dissatisfaction (Uchôa et al., 2019; Marques et al., 2022). A large collection of research has shown a positive correlation between social media usage and low self-esteem (Bessenoff, 2006; Valkenburg et al., 2006; Santarossa and Woodruff, 2017), body dissatisfaction (Fardouly et al., 2015; Grogan, 2016; Holland and Tiggemann, 2016; Cohen et al., 2017; Hendrickse et al., 2017; Santarossa and Woodruff, 2017; Hogue and Mills, 2019; Revranche et al., 2022), eating disorders (Hummel and Smith, 2015; Holland and Tiggemann, 2016; Mingoia et al., 2017; He and Yang, 2022) and other psychological issues such as depression (Twenge et al., 2018; Leimonis and Koutra, 2022).

The harmful impact of exposure to media images on women’s body satisfaction is often attributed to social comparison (Levine and Murnen, 2009). Social comparison theory, as proposed by Festinger in 1954, asserts that humans have an innate tendency to assess their abilities and characteristics by comparing themselves to others, preferably those who are more similar to them, especially when there is no actual means of evaluation. Festinger (1954) delineated two primary forms of social comparison: ‘Downward comparison’ in which individuals compare themselves to those they perceive as less fortunate in some aspect, generally leading to improved mood and feelings of personal worth (Wills, 1991); and ‘Upward comparison’ in which individuals compare themselves to those they perceive as socially superior, typically resulting in negative mood and a potential threat to self-esteem (Gibbons and Gerrard, 1989; Wheeler and Miyake, 1992; Nerini et al., 2009).

Therefore, when applying this theory to social media, a space saturated with idealized images and beauty standards, individuals often find themselves comparing their own physical appearance to these models. This ‘upward’ comparison frequently leads to a self-perception of inadequacy, resulting in a negative evaluation of one’s physical appearance. As a consequence, this is reflected in the subsequent increase in levels of body dissatisfaction (Thompson et al., 1999; Tiggemann and McGill, 2004; Tiggemann and Slater, 2004; Nerini et al., 2009).

Social Networks are platforms, where people are exposed to content related to physical appearance, and excessive use of these is associated with a negative body image among adolescents (Revranche et al., 2022). This association derives from the internalization of the concept of the ideal body presented on the internet, self-objectification, peer feedback and social comparison (Revranche et al., 2022). A key feature that distinguishes contemporary social media from traditional media is its interactivity (Pempek et al., 2009). The active social behaviors in the form of “likes” and “comments” can be generated by Social Networking Site (SNS) users to represent acceptance and can be employed as indicators of popularity (Jong and Drummond, 2013). SNS is a place full of peers’ images and celebrities that create opportunities for social comparisons and internalization determinants of a “social definition of beauty” (Santarossa and Woodruff, 2017). This internalized ideal can become problematic because media content often does not reflect reality: as a result, incomplete, inaccurate and “misrepresented” issues are promoted (Geraee et al., 2015; Grogan, 2016).

Several studies (Holland and Tiggemann, 2016; Murray et al., 2016; Lonergan et al., 2019) identified specific aspects of SNS use related to body dissatisfaction and eating disorders among adolescents and young adults. Recent research has shown that exposure to photos on a platform called “Instagram” depicting the ideal of thinness (compared to images of plus-size women) increases women’s body dissatisfaction (Tiggemann et al., 2018). The use of filters or other digital alteration techniques represents another important photo-based activity that needs closer attention (Holland and Tiggemann, 2016). It also was found that who share modified photos (selfies) on social media are related to the overestimation of body shape and weight, body dissatisfaction, dietary restriction, and internalization of the ideal of thinness (McLean et al., 2015).

The Sociocultural Attitudes Toward Appearance Questionnaire third version (SATAQ - 3) is a tool for evaluating sociocultural pressure and internalization of the beauty standard (Heinberg et al., 1995). This instrument was developed by Heinberg, Thompson and Stormer in its first version in 1995 (Thompson et al., 2004) and has been updated several times (Thompson et al., 2004; Schaefer et al., 2015). The SATAQ, and its later versions, had psychometric qualities tested in populations from many different countries such as Spain, Iran, Brazil, India, France and Italy (Stefanile et al., 2011; Llorente et al., 2015; Rodgers et al., 2016; Neves et al., 2020; Sahlan et al., 2020; Lewis-Smith et al., 2021). Measuring internalization of thin ideal has received a great deal of attention because of the potential risk developing of an eating disorder (Warren and Akoury, 2020; Jankauskiene and Baceviciene, 2022; Negi et al., 2022). In 2011, Stefanile and colleagues validated the Italian version of the questionnaire obtaining good psychometric properties (factor loading, Cronbach alphas and convergent validity) (Stefanile et al., 2011). In 2019 the same authors validated the SATAQ-4-Revised which provides two versions (for female and male population) adapted to the new ideal of beauty that assesses the desire for a muscular and athletic physique (Stefanile et al., 2019).

According to Uchôa and colleagues, the SATAQ-3 measures the influence of traditional media such as television, cinema, and magazines. However, it does not evaluate the impact of the internet and social networks, which have become increasingly significant in the lives of adolescents (Uchôa et al., 2019). Furthermore, even the SATAQ-4 does not provide a comprehensive assessment of the influence of social networks, as it broadly examines the three components of the tripartite model of influence (peer, family, and media), dedicating only four specific questions to the “media” (Schaefer et al., 2015). To the best of our knowledge, no questionnaire in Italian language evaluates the sociocultural pressure and internalization of the beauty standard regardless of gender and based on social media.

Starting from the validation of the questionnaire SATAQ-SM in the female population (aim of this study), we plan to validate it also in the male population and finally in the general one in the next future.

## Materials and methods

### Questionnaires description

The Sociocultural Attitudes Towards Appearance Questionnaire, third version (SATAQ-3), is a self-administered questionnaire composed of 30 items. It was originally developed and validated by Professor Kevin J. Thompson and his colleagues in 2004. An Italian version of the questionnaire was subsequently translated and validated by Stefanile and colleagues in 2011. Professor Kevin J. Thompson, the creator of the SATAQ-3, has granted permission for its use in our current study.

The compiler can indicate their accordance with each item with a Likert scale from 1 (“Strongly disagree”) to 5 (“Strongly agree”). Eight items are reverse scored (3, 6, 9, 12, 13, 19, 27, 28). The total score of mass media influence (with a range from 30 to 150) was classified as: little influence (≤77 points), moderate influence (78–94 points) and great influence (≥95 points) (Uchôa et al., 2019).

The questionnaire is composed of four subscales:

- the *information* subscale consisted of nine items (1, 5, 9, 13, 17, 21, 25, 28, 29) with a range between 9 and 45. It measures the recognition of the social importance of beauty ideals in the mass media (e.g., *TV programs are an important source of information about fashion and “being attractive”*).
- the *pressure* subscale consisted of seven items (2, 6, 10, 14, 18, 22, 26) with a range between 7 and 35. Items indicate a subjective pressure from exposure to media images in order to change my own appearance (e.g., *I’ve felt pressure from TV or magazines to lose weight*).
- the *internalization general* subscale consisted of nine items (3, 4, 7, 8, 11, 12, 15, 16, 27) with a range between 9 and 45. It evaluates the internalization of the beauty ideal promoted by mass media (e.g., *I compare my body to the bodies of TV and movie stars*).
- the *internalization athlete* subscale consisted of five items (19, 20, 23, 24, 30) with a range between 5 and 25. It measures the internalization of the sports ideal (e.g., I try to look like sports athletes; I wish I looked as athletic as sports stars).

The score is given by the sum of the responses to each item, the higher the score the greater the internalization of the specific media message. The Cronbach’s alpha for the SATAQ-3 validated in Italian was uniformly high for the 4 subscales: Information = 0.91, Pressure = 0.91, Internalization-General = 0.94 and Internalization-Athletics = 0.84 (Stefanile et al., 2019).

For our research, we used the Italian version of SATAQ-3 as a starting point, which had been validated by Stefanile and colleagues in 2011. Subsequently, to create the SATAQ-SM questionnaire, we made specific modifications to customize it for use in the context of social media. For example, we made several adjustments to the questionnaire to reflect contemporary media trends. Terms such as “TV programs,” “TV or magazines,” and “TV” were replaced with “social networks” or “websites.” Additionally, “TV and movie stars” was updated to “influencers.” To account for the prevalence of series available on various platforms, we added “series” to items 11 and 25. Furthermore, we removed “on TV” from item 9, considering that music videos are now accessible through other platforms like YouTube and Apple Music. Items 15, 20, 23, 24, 29, and 30 remained unchanged, as they did not contain any of the aforementioned terms (see Table 1).

**Table 1:** Comparison of the three different questionnaires: SATAQ-3, SATAQ-3 Italian version and SATAQ-SM.

**Item number**	**SATAQ-3**	**SATAQ-3 (Italian version)**	**SATAQ-SM**
1	*TV programs* are an important source of information about fashion and “being attractive.”	I *programmi televisivi* sono un’importante fonte riguardo a ciò che è di moda e a ciò che è attraente.	I *social network* sono un’importante fonte riguardo a ciò che è di moda e a ciò che è attraente.
2	I’ve felt pressure from *TV or magazines* to lose weight.	Ho sentito la pressione della *TV o delle riviste* a perdere il peso.	Ho sentito la pressione *dei social network* a perdere il peso.
3	I do not care if my body looks like the body of people who are on *TV*.	Non mi interessa se il mio corpo assomiglia a quello delle persone *in TV.*	Non mi interessa se il mio corpo assomiglia a quello delle persone *sui social network*.
4	I compare my body to the bodies of people who are on *TV*.	Confronto il mio corpo con quello delle persone *in TV*.	Confronto il mio corpo con quello delle persone *sui social network*.
5	*TV commercials* are an important source of information fashion and “being attractive.”	Gli *annunci pubblicitari televisivi* sono un’importante fonte riguardo a ciò che è di moda e a ciò che è attraente.	Gli *annunci pubblicitari nei social network* sono un’importante fonte riguardo a ciò che è di moda e a ciò che è attraente.
6	I do not feel pressure from *TV or magazines* to look pretty.	Non ho sentito la pressione *della TV e delle riviste* ad essere bell*.	Non ho sentito la pressione *dei social network* ad essere bell*.
7	I would like my body to look like the models who appear in *magazines*.	Vorrei che il mio corpo assomigliasse ai modelli che compaiono *sulle riviste*.	Vorrei che il mio corpo assomigliasse ai modelli che compaiono *sui social network*.
8	I compare my appearance to the appearance of *TV and movie* stars.	Confronto il mio aspetto con quello di *star televisive e cinematografiche*.	Confronto il mio aspetto con quello *degli/delle influencer*.
9	Music videos on *TV* are not an important source of information about fashion and “being attractive.”	I *video musicali in TV* non sono un’importante fonte riguardo a ciò che è di moda e a ciò che è attraente.	I *video musicali* non sono un’importante fonte riguardo a ciò che è di moda e a ciò che è attraente.
10	I’ve felt pressure from *TV and magazines* to be thin.	Ho sentito la pressione *della TV e delle riviste* ad essere magr*.	Ho sentito la pressione *dei social network* ad essere magr*.
11	I would like my body to look like the people who are in *movies*.	Vorrei che il mio corpo assomigliasse alle persone *nei film*.	Vorrei che il mio corpo assomigliasse alle persone *nei film e nelle serie televisive*.
12	I do not compare my body to the bodies of people who appear in *magazines*.	Non confronto il mio corpo con quello delle persone che appaiono *nelle riviste*.	Non confronto il mio corpo con quello delle persone che appaiono *nei siti internet*.
13	*Magazine articles* are not an important source of information about fashion and “being attractive.”	Gli *articoli delle riviste* non sono un’importante fonte riguardo a ciò che è di moda e a ciò che è attraente.	*Gli articoli dei siti internet* non sono un’importante fonte riguardo a ciò che è di moda e a ciò che è attraente.
14	I’ve felt pressure from *TV or magazines* to have a perfect body.	Ho sentito la pressione *della TV o delle riviste* ad avere un corpo perfetto.	Ho sentito la pressione *dei social network* ad avere un corpo perfetto.
15	I wish I looked like the models in *music videos*.	Desidero apparire come il/le modell* nei *video musicali*.	Desidero apparire come il/le modell* nei *video musicali*.
16	I compare my appearance to the appearance of people in *magazines*.	Confronto il mio aspetto con quello delle persone *nelle riviste*.	Confronto il mio aspetto con quello delle persone *nei siti internet*.
17	*Magazine advertisements* are an important source of information about fashion and “being attractive.”	La *pubblicità nelle riviste* è un’importante fonte di informazione riguardo a ciò che è di moda e a ciò che è attraente.	*La pubblicità nei siti internet* è un’importante fonte di informazione riguardo a ciò che è di moda e a ciò che è attraente.
18	I’ve felt pressure from *TV or magazines* to diet.	Ho sentito la pressione *della TV o delle riviste* ad intraprendere una dieta.	Ho sentito la pressione *dei social network* ad intraprendere una dieta.
19	I do not wish to look as athletic as the people in *magazines*.	Non desidero sembrare una persona atletica tanto quanto le persone *sulle riviste*.	Non desidero sembrare una persona atletica tanto quanto le persone *sui social network*.
20	I compare my body to that of people in “good shape.	Confronto il mio corpo con quello delle persone “in forma.”	Confronto il mio corpo con quello delle persone “in forma.”
21	Pictures in *magazines* are an important source of information about fashion and “being attractive.	Le immagini *sulle riviste* sono un’importante fonte riguardo a ciò che è di moda e a ciò che è attraente.	Le immagini *sui siti internet* sono un’importante fonte riguardo a ciò che è di moda e a ciò che è attraente.
22	I’ve felt pressure from *TV or magazines* to exercise.	Ho sentito la pressione *della TV o delle riviste* ad allenarmi.	Ho sentito la pressione *dei social network* ad allenarmi.
23	I wish I looked as athletic as sports stars.	Desidero sembrare atletic* tanto quanto le stelle dello sport.	Desidero sembrare atletic* tanto quanto le stelle dello sport.
24	I compare my body to that of people who are athletic.	Confronto il mio corpo con quello delle persone atletiche.	Confronto il mio corpo con quello delle persone atletiche.
*25*	*Movies* are an important source of information about fashion and “being attractive.”	*I film* sono un’importante fonte riguardo a ciò che è di moda e a ciò che è attraente.	*I film e le serie televisive* sono un’importante fonte riguardo a ciò che è di moda e a ciò che è attraente.
26	I’ve felt pressure from *TV or magazines* to change my appearance.	Ho sentito la pressione *della TV o delle riviste* a cambiare il mio aspetto.	Ho sentito la pressione *dei social network* a cambiare il mio aspetto.
27	I do not try to look like the people on *TV*.	Non provo ad assomigliare alle persone *in TV*.	Non provo ad assomigliare alle persone *sui social network*.
28	*Movie starts* are not an important source of information about fashion and “being attractive.”	Le *star del cinema* non sono un’importante fonte riguardo a ciò che è di moda e a ciò che è attraente.	*Gli influencer* non sono un’importante fonte riguardo a ciò che è di moda e a ciò che è attraente.
29	Famous people are an important source of information about fashion and “being attractive.”	Le persone famose sono un’importante fonte riguardo a ciò che è di moda e a ciò che è attraente.	Le persone famose sono un’importante fonte riguardo a ciò che è di moda e a ciò che è attraente.
30	I try to look like sports athletes.	Tento di assomigliare agli atleti sportivi.	Tento di assomigliare agli atleti sportivi.

*In the Italian language, the asterisk is used to avoid indicating the gender to which the word refers.

These modifications were made collaboratively by three native Italian-speaking researchers. The decision to revise the questionnaire using terms related to social media is justified by the profound impact these platforms have had on the communication landscape (Twenge et al., 2019). Unlike social media, which has become the predominant and easily accessible means of reaching a wider audience, traditional media outlets have experienced a decline in influence due to the sway of the internet and social media (Al-Quran, 2022).

*Rosenberg self-esteem scale (RSES)* was developed in 1965 by Morris Rosenberg (Rosenberg, 1965). It is the most widely used rating scale to measure an individual’s self-esteem level for research purposes, although not for diagnostic purposes, in the case of psychological issues. It was translated and validated in Italian in 1997 (Prezza et al., 1997). The RSES is a self-administrated questionnaire, consisting of 10 statements and respondents are asked to rate their level of agreement with each statement on a Likert scale from 1 (“Strongly disagree”) to 4 (“Strongly agree”). Five items are reverse scored (3, 5, 8, 9, 10). The total score range is from 0 to 30. Scores between 15 and 25 are in the normal range; scores below 15 indicate low self-esteem.

The *Eating Attitudes Test* (EAT-26) is probably the most widely used standardized measure of symptoms and concerns characteristic of eating disorders (Garner and Garfinkel, 1979; Garner et al., 1982). The EAT-26 does not yield a specific diagnosis of an eating disorder. It was translated and validated in Italian in 1988 (Cuzzolaro and Petrilli, 1988). The EAT-26 is a self-administrated questionnaire, consisting of 26 statements and respondents are asked to rate their level of agreement with each statement on a 6-point Likert scale from “Always” to “Never.” One item is reversed score (25). The questionnaire consists of three subscales: dieting (defined by items 1, 6, 7, 10, 11, 12, 14, 16, 17, 22, 23, 24, and 25), Bulimia (3, 4, 9, 18, 21, and 26) and oral control (2, 5, 8, 13, 15, 19, and 20). The total score ranges from 0 to 75 (symptoms of anorexia or bulimia are unlikely if the score is <20; symptoms of anorexia or bulimia are likely if the score is >20). The Dieting subscale has a range between 0 and 39 while the other two (Bulimia and Oral against) between 0 and 21. The higher the score, the greater the presence of the symptom.

### Sampling and recruitment

The SATAQ-SM was uploaded to Microsoft Forms, an online survey creation platform, and distributed via a shared link on social media platforms such as WhatsApp and Instagram from February 19, 2021, to April 8, 2021. On WhatsApp, individuals were asked to complete the questionnaire and share it with others, while on Instagram, we used ‘stories’ to encourage participation by providing a clickable link to our survey. No specific website niches, influencers, or blogs were used for distribution; it was solely carried out through the contacts of AMR, LP, and SC.

Clark and Watson (1995) suggested that an adequate sample size for questionnaire validation should be no less than 300 respondents while Comrey and Lee (1992) proposed a graded scale of sample size: 100 respondents = poor; 200 = fair; 300 = good; 500 = very good; ≥1,000 = excellent. The goal was to achieve several compilations of no less than 400 to satisfy both Comrey and Lee (1992) and Clark and Watson (1995) sample size criteria. We decided to include all participants who completed all the questions. The only inclusion criterion was reading the study’s objectives and methods and providing informed consent. No grants or financial incentives were provided to participants in the study. A total of 485 females and 74 males chose to participate in the research by responding to the questionnaire. The authors chose not to consider the male subpopulation due to its limited size, while the female population represents a sufficient and highly interesting sample for the study.

### Statistical analysis

Descriptive statistics (mean and standard deviation for quantitative data, absolute and percentual frequency for qualitative data) were computed for socio-demographic variables.

We analyzed scale structure using confirmatory factor analysis which investigates whether the empirical data fits a specified theoretical model – in this case the four-factor structure – defined by Thompson et al. (2004) and Stefanile et al. (2011). To ensure there was a sufficient association between items and factors to justify a CFA, the following correlations were inspected: inter-item (the average correlation should be between 0.20 and 0.40; Piedmont, 2014), item-to-factors (coefficient > 0.4, suggests convergent validity of items within the same factors), and inter-factors (coefficient of >0.4 support convergent validity). The normal distribution of both the individual items and the subscales was also verified by analyzing the skewness and kurtosis values (values beyond −2 and +2 are considered indicative of substantial nonnormality according to Hair et al., 2022).

Assessment of goodness-of-fit was based on standard model fit criteria (Kline, 2005). χ^2^ was not used as is too sensitive to sample size: a relative χ^2^ value (χ^2^/df) less than 5 is considered a common benchmark. Additional fit indices were examined, including the Root Mean-Squared Error of Approximation (RMSEA; <0.08 is considered adequate fit; Hu and Bentler, 1999); Comparative Fit Index (CFI) (Bentler, 1990), and Tucker–Lewis Index (TLI) (Tucker and Lewis, 1973) with a cut-off of ≥0.95 indicating good for the latter two fit criteria (Hu and Bentler, 1999).

Internal consistency was assessed using McDonald’s omega (ω) (an omega coefficient of 0.70 or greater was considered acceptable) (Kalkbrenner, 2023).

Criterion validity was calculated by correlating the SATAQ factors scores with the total RSES scale and the EAT-26 total score. We can hypothesize: an inverse association between the scores of SATAQ-SM and RSES; a direct association between the scores of SATAQ-SM and EAT-26.

## Results

The questionnaire was disseminated through social media, allowing anyone to participate voluntarily. We chose to include only women in our analysis as their numbers were higher compared to other genders (Women = 485; Men = 74); therefore, it would not have been possible to generalize the results to the entire population (Comrey and Lee, 1992; Clark and Watson, 1995). Their socio-demographic characteristics are summarized in Table 2. Most of the participants were under the age of 26 (*N* = 383; 78.97%). Moreover, 45.98% (*N* = 223) of the females were engaged or in a stable affective relationship. Also, 50.11% (*N* = 243) had a high school diploma while 28.04% (*N* = 136) had a middle school diploma.

**Table 2:** Sociodemographic characteristics of the sample.

	*n*	%
**Age**		
Less than 18 years old	120	24.74
18–21	148	30.52
22–25	115	23.71
26–30	46	9.48
31–40	32	6.60
Over 40 years old	24	4.95
**Civil status**		
Single	171	35.26
Engaged/Stable affective relationship	223	45.98
Married/Civil Union	31	6.39
Separated/Divorced	5	1.03
Other	55	11.34
**Level of education**		
Elementary school	2	0.41
Middle school	136	28.04
High school	243	50.11
Bachelor/Master’s degree	104	21.44

### Psychometric properties

**Figure 1:**
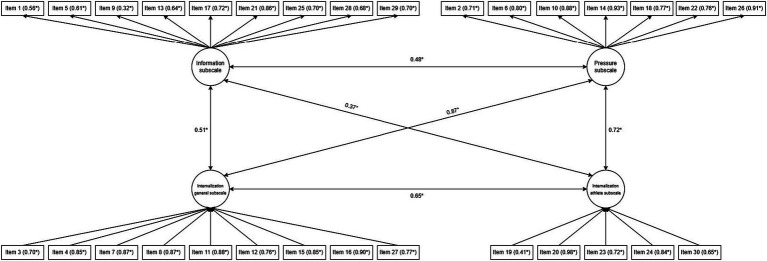
Model plot of the 4-factor structure of the questionnaire.

The average inter-item correlation is described in Table 3: for two factors (Information and Internalization-Athlete) the average value obtained falls within the indices suggested by the literature while for the other two (Pressure and Internalization-General) they are higher. Item-to-factors correlations (Table 4) are all greater than or equal to 0.40 except for items 9 (Factor 1; 0.35) and 19 (Factor 4; 0.34). Finally, with regards to inter-factors correlation analysis, only that between Factor 1 (Information) and Factor 4 (Internalization-Athlete) was slightly below the reference cut-off: 0.37 (Table 5 and Figure 1).

**Table 3:** Inter-item correlation analysis.

**Factor 1 (Information)**		**Factor 2 (Pressure)**		**Factor 3 (Internalization-General)**		**Factor 4 (Internalization-Athlete)**	
**Average mean**	**Number of items**	**Average mean**	**Number of items**	**Average mean**	**Number of items**	**Average mean**	**Number of items**
0.37	9	0.60	7	0.61	9	0.43	5

**Table 4:** Item-to-factors correlation analysis.

**Factor 1 (Information)**		**Factor 2 (Pressure)**		**Factor 3 (Internalization-General)**		**Factor 4 (Internalization-Athlete)**	
**Item**	**Correlation**	**Item**	**Correlation**	**Item**	**Correlation**	**Item**	**Correlation**
1	0.46	2	0.66	3	0.64	19	0.34
5	0.53	6	0.66	4	0.78	20	0.48
9	0.35	10	0.81	7	0.80	23	0.66
13	0.55	14	0.83	8	0.80	24	0.75
17	0.63	18	0.71	11	0.80	30	0.64
21	0.72	22	0.69	12	0.69		
25	0.56	26	0.82	15	0.77		
28	0.57			16	0.80		
29	0.59			27	0.71		

**Table 5:** Inter-factors correlation analysis.

	**Factor 2 (Pressure)**		**Factor 3 (Internalization-General)**		**Factor 4 (Internalization-Athlete)**	
	Estimate	*p*	Estimate	*p*	Estimate	*p*
Factor 1 (Information)	0.48	<0.001	0.51	<0.001	0.37	<0.001
Factor 2 (Pressure)			0.87	<0.001	0.72	<0.001
Factor 3 (Internalization-General)					0.65	<0.001

### Confirmatory factor analysis

The skewness and kurtosis indices are all within the limits defined by the literature (−2 and +2). The four-factor model appears to be supported by the fit indices described in Table 6: RMSEA, an absolute measure of fit based on the non-centrality parameter, is 0.07 (reference value: <0.008); CFI, which assesses model fit by examining the discrepancy between the data and the hypothesized model, is highly favorable at 0.99 (reference value: >0.95), and TLI, a statistical measure used to evaluate goodness of fit, also exceeds the expected cutoff at 0.99 (reference value: >0.95).

**Table 6:** Fit indices of the confirmatory factor analysis.

**Index**	**Value**	**Reference value**
χ^2^/df	3.73	<5
RMSEA	0.07	<0.08
Comparative Fit Index (CFI)	0.99	>0.95
Tucker-Lewis Index (TLI)	0.99	>0.95

As described in Table 7 and Figure 1, all the items defining the four factors had a factor loading ≥0.40.

**Table 7:** Factor loadings in the 4 factors model.

**Factor**	**Item**	**Estimate**	***z*-value**	***p***	**95% Confidence interval**	
					**Lower bound**	**Upper bound**
Factor 1 (Information)	1	0.56	40.09	<0.001	0.54	0.59
	5	0.61	45.36	<0.001	0.58	0.64
	9(R)	0.32	22.21	<0.001	0.29	0.35
	13(R)	0.64	48.35	<0.001	0.62	0.67
	17	0.72	56.29	<0.001	0.70	0.75
	21	0.86	67.11	<0.001	0.84	0.89
	25	0.70	51.17	<0.001	0.67	0.73
	28(R)	0.68	49.73	<0.001	0.65	0.70
	29	0.70	53.53	<0.001	0.67	0.72
Factor 2 (Pressure)	2	0.71	80.94	<0.001	0.70	0.73
	6(R)	0.80	96.23	<0.001	0.79	0.82
	10	0.88	121.93	<0.001	0.87	0.90
	14	0.93	133.87	<0.001	0.91	0.94
	18	0.77	88.30	<0.001	0.75	0.79
	22	0.76	88.52	<0.001	0.75	0.78
	26	0.91	127.09	<0.001	0.90	0.92
Factor 3 (Internalization-General)	3(R)	0.70	83.29	<0.001	0.68	0.71
	4	0.85	117.58	<0.001	0.83	0.86
	7	0.87	129.23	<0.001	0.86	0.88
	8	0.87	129.49	<0.001	0.86	0.88
	11	0.88	129.94	<0.001	0.87	0.89
	12(R)	0.76	97.29	<0.001	0.75	0.78
	15	0.85	119.19	<0.001	0.84	0.87
	16	0.90	134.76	<0.001	0.88	0.91
	27(R)	0.77	98.44	<0.001	0.76	0.79
Factor 4 (Internalization-Athlete)	19(R)	0.41	33.32	<0.001	0.39	0.44
	20	0.98	79.13	<0.001	0.96	1.01
	23	0.72	62.33	<0.001	0.70	0.74
	24	0.84	76.90	<0.001	0.82	0.86
	30	0.65	55.75	<0.001	0.63	0.68

### Internal consistency

The McDonald’s coefficient omega value for the entire questionnaire was 0.95. All four subscales have obtained values significantly above the reference cut-off (>0.70): Internalization-General: 0.94; Information: 0.84; Internalization-Athlete: 0.81; Pressures: 0.90 (Table 8). The values if a specific item were deleted are also shown in Table 8, and there are no solutions for a potential increase in reliability values.

**Table 8:** Internal consistency analysis.

Items	Internalization-General		Information		Internalization-Athlete		Pressures		Total score	
	ω*	ω *if item deleted*	ω	ω *if item deleted*	ω	ω *if item deleted*	ω	ω *if item deleted*	ω	ω *if item deleted*
1	0.94		0.84		0.81		0.92		0.95	
2								0.91		0.95
3		0.93								0.95
4		0.93								0.94
5				0.83						0.95
6								0.91		0.94
7		0.93								0.94
8		0.92								0.94
9				0.83						0.95
10								0.90		0.94
11		0.92								0.94
12		0.93								0.94
13				0.84						0.95
14								0.90		0.94
15		0.93								0.94
16		0.93								0.94
17				0.82						0.95
18								0.91		0.95
19						0.83				0.95
20						0.80				0.94
21				0.81						0.95
22								0.91		0.94
23						0.76				0.95
24						0.71				0.95
25				0.82						0.95
26								0.90		0.94
27		0.93								0.94
28				0.82						0.95
29				0.82						0.95
30						0.76				0.95

* McDonald’s omega.

### Criterion validity

The correlations between the SATAQ-SM (factors scores) with the RSES and EAT-26 are all statistically relevant (*p* < 0.001) as described in Table 9. There is a statistically negative correlation between the four factors of SATAQ-SM and RSES total score while there is a positive association between the scores of the SATAQ-SM subscales and the total score of the EAT-26.

**Table 9:** Correlations between the SATAQ-SM (factors scores) with the RSES total score.

		SATAQ-SM			
		Information	Pressures	Internalization General	Internalization Athlete
Rosenberg self-esteem scale (Total score)	*r*	−0.139	−0.375	−0.458	−0.240
	*p*	0.001	<0.001	<0.001	<0.001
	*N*	565	565	565	565
Eating Attitude Test (Total score)	*r*	0.127	0.388	0.381	0.271
	*p*	0.003	<0.001	<0.001	<0.001
	*N*	485	485	485	485

## Discussion

The aim of the study was to validate the SATAQ-SM. The original SATAQ-3 consists of four subscales: Information, Pressures, Internalization-General and Internalization-Athlete. The factorial analysis conducted on the SATAQ-SM replicated the patterns of item loadings found in published studies using the original version of SATAQ-3.

Furthermore, the McDonald Omega values suggest excellent internal consistency, both for the four subscales and for an overall composite. These consistency levels are similar to those of the original version evaluated with Cronbach’s Alpha (Thompson et al., 2004). For example, the internal consistency for the Pressures subscale was 0.92 for SATAQ-SM and 0.92 for SATAQ-3. The value for the total subscale was 0.95 for SATAQ-SM and 0.96 for SATAQ-3.

Moreover, each item correlates with the subscale to which it belongs (factor loading). The inter-factor correlation, at 0.37, falls just slightly below 0.40 for Factor 1 (Information) and Factor 4 (Internalization-Athlete). Nonetheless, this correlation remains statistically significant. Based on the existing literature, we can affirm that these findings do not adversely affect the psychometric properties of the instrument.

These findings allow us to hypothesize that social media now assume a role akin to television and magazines in contemporary society. As demonstrated in literature, digital media has, in fact, taken over the position once held by television as the foremost source of news in many nations (Majo-Vazquez and González-Bailón, 2018). Furthermore, another study emphasizes the substantial embrace of social media by adolescents, resulting in a considerable decline in the consumption of conventional television programs and magazines (Twenge et al., 2019). This study goes on to highlight that the widespread integration of social media, especially among the iGen generation, has led to a marked decrease in the utilization of traditional TV programs and magazines (Twenge et al., 2019).

Additionally, correlation analysis between EAT-26 and SATAQ-SM revealed a noteworthy relationship. The findings indicate that there is an association between symptoms of eating disorders and the influence of social media on individuals’ perceptions of self. This correlation outcomes aligns with a recent systematic review showing that the misuse or intensive use of social media is linked to a range of negative effects including body dissatisfaction, low self-esteem, and notably, an increased likelihood of developing eating disorders (Vincente-Benito and Ramírez-Durán, 2023).

Finally, the correlation between RSES and SATAQ-SM shows that with an increase in self-esteem, the influence of social media on the perception of self is reduced. Recent evidence suggests that high self-esteem protects adolescents against the internalization of appearance ideals (Espinoza et al., 2019). Adolescence is a period of identity formation, self-expression and most important peer acceptance (Jankauskiene and Baceviciene, 2022). Media use is an important agent for online and offline developmental issues for adolescents (Gerwin et al., 2018).

However, despite the results discussed above, this study has several limitations. Firstly, the validation is based on an opportunistic female sample, and the results cannot be generalized to the entire general population. Further studies, not limited to cross-sectional designs, are needed to determine whether the questionnaire is adaptable to different contexts.

Secondly, the distribution of age groups is different from the actual Italian female population (under 25: 78.97% vs. 22.16%) (Istituto Nazionale di Statistica, 2023). Therefore, our sample is not adequately representative of all ages, so it may be optimal to validate the SATAQ-SM on specific age groups. On the other hand, there is evidence that the age group most actively using social media falls between 20 and 29 years old (We Are Social and Meltwater, 2023), a demographic well-represented in our sample. In future studies, it might be possible to address these limitations by administering the questionnaire in specific settings, such as educational projects at various schooling levels or within universities. Providing the questionnaire in locations frequented by older individuals could help capture a wider range of responses and mitigate potential age-related bias in social media use.

Nevertheless, in future studies, it might be possible to mitigate these limitations by administering the questionnaire in specific settings, such as educational projects at various levels of schooling or within universities. Providing the questionnaire in locations frequented by older individuals could help capture a broader range of responses and address potential age-related biases in social media use.

Third, since this study protocol did not have a test–retest analysis we could not determine the temporal stability of responses.

Despite these limitations, we believe that the current psychometric evidence provides support for using SATAQ-SM in research. Investigating the influence of social media on body image perception is a field with broad implications (Artoni et al., 2021). It has been a recent phenomenon, where social media has become an integral part of our everyday life. Hence, the researcher still has much to discover about the effects of social media on people.

In fact, the use of social media significantly impacts mental health. When used correctly, it can enhance connections, increase self-esteem, and improve a sense of belonging (Zsila and Reyes, 2023). Additionally, social media provide opportunities to enhance support for users by facilitating social connections. For instance, online communities offer spaces for discussions about health conditions, adverse life events, or everyday challenges (Ulvi et al., 2022).

Creating tools like the SATAQ-SM, which assess social media influence, can contribute to the prevention of mental health problems (Riehm et al., 2019). It’s important to note that demonizing platforms like Instagram and other social media is not productive. Different individuals use social media in various ways. Therefore, we can approach the experience of social media more consciously (Campisi et al., 2015).

## Conclusion

This study attempts to validate SATAQ-SM, a translated and modified version of SATAQ-3 for women. The McDonald Omega values indicate excellent internal consistency for both the four subscales and the overall composite. These consistency levels are comparable to those of the original version, which was assessed using Cronbach’s Alpha (Thompson et al., 2004). The high data quality can be attributed to the well-designed original SATAQ-3 questionnaire. This adapted Italian version questionnaire could assist in investigating groups at greater risk for developing eating and body image disturbances in relation to social media use.

Regardless of the limitations explained previously, we believe that SATAQ-SM is an exciting research tool for future exploration on understanding the complex connection between social media usage and body image perception.

## Data availability statement

The raw data supporting the conclusions of this article will be made available by the authors, without undue reservation.

## Ethics statement

Ethical approval was not required for the studies involving humans in accordance with the local legislation Regione Emilia – Romagna AOU 0007852/20 (16/03/2020) and the Comitato Etico Area Vasta Emilia Nord (AVEN) requirements. The risk of identity theft is low, and the questionnaires administered were directed at the general population without generating a diagnosis or allowing the definition of psychopathological conditions. The studies were conducted in accordance with the local legislation and institutional requirements. Written informed consent for participation was not required from the participants or the participants’ legal guardians/next of kin in accordance with the local legislation Regione Emilia – Romagna AOU 0007852/20 (16/03/2020) and the Comitato Etico Area Vasta Emilia Nord (AVEN) requirements. All the participants provided written informed consent.

## Author contributions

All authors listed have made a substantial, direct, and intellectual contribution to the work and approved it for publication.
